# Statistical and Dynamical Properties of Topological Polymers with Graphs and Ring Polymers with Knots

**DOI:** 10.3390/polym9070252

**Published:** 2017-06-28

**Authors:** Tetsuo Deguchi, Erica Uehara

**Affiliations:** Department of Physics, Faculty of Core Research, Ochanomizu University, Ohtsuka 2-1-1, Bunkyo-ku, Tokyo 112-8610, Japan; uehara.erika@ocha.ac.jp

**Keywords:** polymer, topology, knot, simulation

## Abstract

We review recent theoretical studies on the statistical and dynamical properties of polymers with nontrivial structures in chemical connectivity and those of polymers with a nontrivial topology, such as knotted ring polymers in solution. We call polymers with nontrivial structures in chemical connectivity expressed by graphs “topological polymers”. Graphs with no loop have only trivial topology, while graphs with loops such as multiple-rings may have nontrivial topology of spatial graphs as embeddings in three dimensions, e.g., knots or links in some loops. We thus call also such polymers with nontrivial topology “topological polymers”, for simplicity. For various polymers with different structures in chemical connectivity, we numerically evaluate the mean-square radius of gyration and the hydrodynamic radius systematically through simulation. We evaluate the ratio of the gyration radius to the hydrodynamic radius, which we expect to be universal from the viewpoint of the renormalization group. Furthermore, we show that the short-distance intrachain correlation is much enhanced for real topological polymers (the Kremer–Grest model) expressed with complex graphs. We then address topological properties of ring polymers in solution. We define the knotting probability of a knot *K* by the probability that a given random polygon or self-avoiding polygon of *N* vertices has the knot *K*. We show a formula for expressing it as a function of the number of segments *N*, which gives good fitted curves to the data of the knotting probability versus *N*. We show numerically that the average size of self-avoiding polygons with a fixed knot can be much larger than that of no topological constraint if the excluded volume is small. We call it “topological swelling”.

## 1. Introduction

### 1.1. Topological Polymers and Knotted Ring Polymers

Polymers of novel knotted structures and those of complex chemical connectivity expressed by graphs have been found in various fields recently, such as those of DNA, proteins and synthetic polymers [1,2,3,4,5,6,7,8]. Statistical and dynamical properties of polymers with nontrivial structures in chemical connectivity and those derived from topological constraints such as ring polymers with fixed knots have attracted much interest in several branches of physics, chemistry and biology. Although some properties of ring polymers in solution were studied rather early, such as in the 1940s and 1960s [9,10,11,12], circular DNA had been found in nature in the 1960s, and knotted DNA molecules are synthesized in experiments in the 1980s [2,13,14,15]; looped or knotted proteins had been found in nature during the 2000s [5,16]. Here, we recall that the three-dimensional conformation of a ring polymer in solution is described by a closed curve with no ends, and its topology is represented by a knot. Several theoretical studies on knotted ring polymers in solution are reviewed in [3,4].

**Figure 1:**
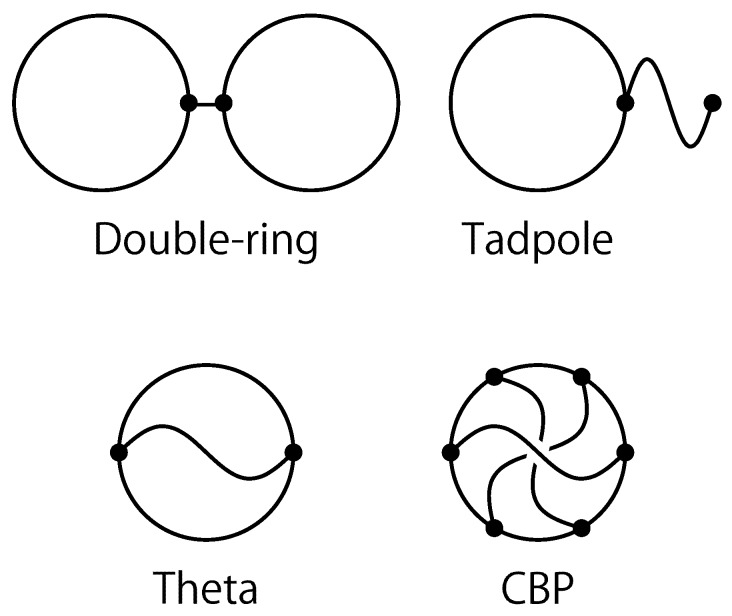
Examples of topological polymers with graphs with their names: ‘tadpole’ for a tadpole or lasso polymer, ‘theta’ for a θ-shaped polymer, ‘double-ring’ for a double-ring, bicyclic or eight-shaped polymer, and ‘CBP’ for a complete bipartite-graph $K_{3,3}$ polymer, respectively. Connecting segments and terminal segments are shown by filled circles.

After novel developments in synthetic chemistry, polymers with various different chemical or topological structures were synthesized in experiments recently during the last decade such as ring polymers, tadpole (or lasso) polymers, double-ring (or di-cyclic) polymers, multiple-ring (or multi-cyclic) polymers and even complete bipartite graph polymers [8,17,18,19,20,21,22,23,24,25]. In Figure 1, some graphs corresponding to simple topological polymers are shown. Lasso polymers are studied in association with protein folding [26]. For multiple-ring (or multi-cyclic) polymers, hydrodynamic properties were theoretically studied by a perturbative method in the 1960s [27]. For double-ring polymers, they are studied by the Monte Carlo (MC) simulation of self-avoiding double-polygons [28]. It seems that topological polymers are also related to ravels as novel entanglements of spatial graphs [29] and non-planar RNA structures corresponding to pseudoknots [30].

In this review, we first present recent theoretical results of topological polymers associated with some graphs and then those of knotted ring polymers in solution. We call polymers with nontrivial structures in chemical connectivity topological polymers. For instance, all of the structural isomers of a given polymer are called topological polymers. Furthermore, we also call polymers with nontrivial topology of spatial graphs as embeddings in three dimensions topological polymers, for simplicity [8,31]. Here, we remark that graphs with no loop such as stars or brushes have only a trivial topology, while graphs with loops such as rings, multiple-rings and theta curves can be topologically nontrivial, for instance, through being knotted in some loops or linked among some loops. Such knotted or linked graphs have a nontrivial topology of spatial graphs as embeddings in three dimensions. For example, nontrivial knots are topologically nontrivial even as spatial graphs embedded in three dimensions.

The semi-flexibility of polymer chains (see, for instance, [32]) plays a crucial role in the topological properties of circular double-stranded DNA chains. They are negatively charged. While the long-range Coulomb repulsive forces are screened by counter ions in solution, the short-range Coulomb repulsions among the segments in a chain make it very stiff. The DNA chains are thus modeled as stiff chains or a sequence of hard cylindrical segments [33]. The effective thickness of DNA chains approximately corresponds to the screening length and can be several times larger than the geometric diameter [34,35]. However, it is much smaller than the statistical length of DNA chains since they are very stiff. Therefore, DNA chains are considered as thin stiff chains [36]. Here, we remark that thin polymer chains have small excluded volume. In the study of random knotting, we introduce a model of self-avoiding polygons (SAP) consisting of impenetrable cylindrical segments with small excluded volume, which is considered as a discrete version of worm-like chains. We call this the cylindrical SAP model.

For the knotting probability, we shall show numerically that topological effects of polymer chains decrease very quickly if the excluded volume increases from zero or a value close to zero even by a very small amount of quantity. We therefore suggest that it should be the reason why knotted ring polymers were synthesized in the experiments of circular DNA for the first time in the history of science that DNA chains are thin stiff polymer chains, and hence, they are easily knotted among themselves. For standard flexible linear or ring polymer chains, however, the probability of being nontrivially knotted is very small since they correspond to self-avoiding walks or polygons with some definite excluded volume.

We thus predict that polymers with nontrivial topology as spatial graphs should be synthesized most easily for semi-flexible polymer chains. Although it seems that only topological polymers consisting of flexible chains have been synthesized so far, we expect that knotted semiflexible topological polymers will be synthesized in the near future.

### 1.2. Knotted Ring Polymers in Solution

**Figure 2:**
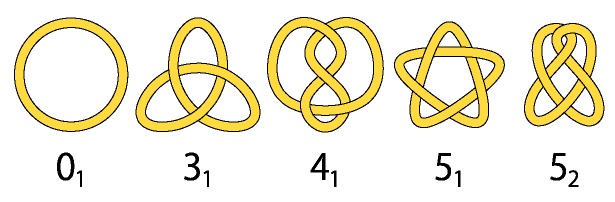
Knot diagrams of the trivial knot $0_{1}$, the trefoil knot $3_{1}$, the figure-eight knot $4_{1}$ and the two prime knots $5_{1}$ and $5_{2}$ whose minimal crossing number is given by five.

Let us briefly review historical developments in the study of topological effects of ring polymers. Some properties of ring polymers in dilute solution were studied theoretically in the 1940s and 1960s [9,10,11,12]. However, no topological effect of ring polymers was considered explicitly at that time. The issue of the topological effects of ring polymers was first addressed in the 1960s in biology by Delbrück [37] associated with replication of DNA double-stranded chains and in chemistry by Frisch and Wasserman [38] associated with isomers derived from different topological structures. It was conjectured by them that if we produce or synthesize long ring polymers in dilute solution, they will be knotted with high probability. Here, we remark that knot diagrams of some knots are shown in Figure 2. They correspond to symbols associated with the minimal number of crossing points in the diagrams [39].

The effects of topological constraints on the statistical properties of ring polymers were explicitly studied through simulation in the pioneering research [40,41] in the 1970s by Vologodskii et al. Here, we remark about some relevant theoretical researches [42,43,44]. Then, the conjecture proposed by Frisch, Wasserman and Delbrück was proven mathematically: it was shown by Sumners, Whittington and Pippenger that every infinitely-long self-avoiding walk is knotted [45,46]. Furthermore, knotted ring polymers were explicitly derived first in DNA experiments [14,15]. Circular DNAs of different topologies are separated into knotted species by gel electrophoresis [14,15,47,48]. The probability of random knotting was measured in DNA experiments [34,35]. It was shown that the concept of tight knots, i.e., ideal knots, plays a central role in the dynamics of ring polymers [49,50,51]. Among the recent progress of DNA experiments, diffusion coefficients of linear, relaxed circular and supercoiled DNAs in solution have been measured quite accurately [52,53] where the DNA double helices are unknotted.

In polymer physics, anomalous osmotic pressure was observed for the solution of ring polymers at the θ temperature of corresponding linear polymers [54]. In order to understand the phenomenon, the topological interaction between a pair of ring polymers was studied [55,56]. Recently, ring polymers of large molecular weights were synthesized not only quite effectively [19], but also with small dispersions and of extreme high purity [20,21,57]; synthetic ring polymers with nontrivial knots have been synthesized [20,57]. Knotted structures are also studied for proteins [5,58,59]. We recall that polymers with various different chemical connectivity are now systematically synthesized [8].

In order to study the topological properties of ring polymers in solution, we introduce random polygons (RP) and self-avoiding polygons (SAP) [60]. In Section 5, we present numerical results on the probability of random knotting [61,62,63,64,65,66,67,68,69,70,71,72,73,74,75,76,77,78,79,80,81] and, in Section 6, the mean-square radius of gyration of knotted ring polymers in solution [43,82,83,84,85,86,87,88,89,90,91,92,93,94,95].

The topological constraint that polygons should have a given knot type restricts the number of possible conformations of the polygons. Consequently, the available volume in the phase space of such a polygon can be much smaller than that of no topological constraint. The rate of reduced volume in the phase space gives the probability of random knotting. We also call it the knotting probability (see Section 5.1). We give a formula expressing the knotting probability as a function of the number of segments *N* (see Section 5.5). Furthermore, we show that the probability for a self-avoiding polygon having a nontrivial knot type decreases if the excluded volume parameter increases (see Section 5.6). It follows that the topological effect in SAP is significant if the excluded volume is small. In the case of the discrete worm-like chains, the effect of topological entanglement is important if the chain thickness is small.

The average size of RP under a topological constraint can be much larger than that of no topological constraint, if the number of nodes *N* is large enough (see Section 6.2). Here, we denote by the average size the mean-square radius of gyration. The enhancement should be a consequence of “repulsive entropic forces” acting among the segments of each polygon under the topological constraint, which were first suggested by des Cloizeaux [43] (see also [83]). We therefore call it topological swelling [85,86]. Topological swelling occurs for SAP with a fixed knot if the excluded volume is small enough and the number of nodes *N* is large enough [85,86]. Thus, the effect of entropic reduction results not only in the knotting probability, but also in the effective repulsions among polygonal segments, so that the average size of random polygons with a fixed knot type enhances.

Other aspects of knotted ring polymers in solution have also been studied. We now summarize them as follows. The two-point correlation functions and the scattering functions for ring polymers under topological constraints have been studied numerically [11,96,97,98,99] and experimentally [44], and the diffusion coefficients of ring polymers with given knot types have been evaluated in simulation [87,100,101,102,103,104]. The probability of random linking was derived in [41,68,105,106,107], which is related to the anomalous osmotic pressure in θ solution [108,109,110]. Furthermore, there are many interesting topics related to the topological effects of ring polymers: the probability of forming a local knot in a random polygon [111,112], flat knots (i.e., knotted ring polymers in two dimensions) [113,114], the mean square radius of gyration of ring polymers in melt [115,116,117,118,119], the minimal step number of a lattice knot [120,121], the relaxation dynamics of a knotted ring polymer in solution [122], etc. [123,124].

### 1.3. Approaches to Topological Polymers with Nontrivial Chemical Connectivity

Let us recall that only recently it has become possible to synthesize polymers of complex chemical connectivity in experiments, which we call topological polymers. It should be thus interesting to study topological polymers by extending the methods in the study of ring polymers with a fixed knot. Here, we also recall that topological polymers associated with spatial graphs as embeddings in three dimensions can be regarded as a generalization of knots into spatial graphs.

We call a polymer in which the structure of chemical connectivity is expressed by a graph *F* a topological polymer of graph *F* and also call it a graph *F* polymer. Furthermore, we call a polymer with the topology of a spatial graph *F* a topological polymer of spatial graph *F* and also a spatial graph *F* polymer [31]. Here, we mean by a spatial graph an embedded graph that exists in a specific position in three-dimensional space [125].

For an illustration, four graphs of topological polymers are shown in Figure 1: tadpole (or lasso), θ-shaped, double-ring and complete bipartite $K_{3,3}$ graphs. We remark that a complete bipartite graph $K_{3,3}$ gives one of the simplest non-planar spatial graphs [125]. Here, we call a graph with the property that no embedding of it in three dimensions can lie in a plane non-planar. The graph of a tadpole, which is also called a lasso, corresponds to a tadpole polymer. In the review, we assume that the circular part and the tail part of a tadpole polymer have the same number of segments. The graph of a ‘theta’ in Figure 1 denotes a θ-shaped curve, which we also call a theta curve. It corresponds to the molecular structure of a θ-shaped polymer. Each branch connecting the two vertices has the same number of segments throughout the present review. The graph of a ‘double ring’ corresponds to the molecular architecture of a double-ring polymer. Here, we construct the tetravalent (i.e., four-valent) vertex with two trivalent vertices connected by one bond between them. We consider that the distance of the two vertices is minimal, so that the whole structure makes a tetravalent vertex. In the review, we assume that each ring has the same number of segments.

It is fundamental to evaluate the mean-square radius of gyration and the diffusion coefficient in order to characterize the physical aspects of topological polymers such as the statistical and dynamical properties. We numerically evaluate them as follows. First, we construct a weighted ensemble of ideal topological polymers by an algorithm, which is based on some properties of quaternions [31,126]. By this algorithm, we generate *N*-step random walks connecting any given two points [126,127], where the computational time is linearly proportional to the step number *N*. We evaluate the statistical average of some physical quantities over the weighted ensemble (see Section 2.1). Here, we mean by an ideal topological polymer a polymer model consisting of ideal chains that have no excluded volume. Second, we construct an ensemble of conformations of real topological polymers by performing the molecular dynamics of the Kremer–Grest model [128] and evaluate the statistical average of the quantities over the ensemble (see Section 2.2). Here, we mean by a real topological polymer a polymer model consisting of real chains that have nonzero excluded volume. Third, by comparing the results of the real topological polymers with those of the ideal ones, we numerically show some common properties of real and ideal topological polymers.

We indeed observe numerically some properties in common for ideal and real topological polymers. For instance, the set of ratios of the root mean-square radius of gyration $R_{G}$ and the hydrodynamic radius $R_{H}$ among different topological polymers is given by almost the same set of values for ideal and real topological polymers if the valency of each vertex is equal to or less than three in the graphs, as far as for the polymers we have investigated (see Section 3.1 and Section 3.2). This agrees with Tezuka’s observation that the results of the size exclusion chromatography (SEC) are not affected by the excluded volume if each vertex of a topological polymer has less than or equal to three connecting bonds [31,129]. Here, we remark that the mean-square radius of gyration $R_{G}^{2}$ and the hydrodynamic radius $R_{H}$ are related to experimental results, such as the SEC spectrum. We evaluate the hydrodynamic radius $R_{H}$ through Kirkwood’s approximation [130,131,132,133].

The ratio of the root mean-square gyration radius $R_{G}$ to the hydrodynamic radius $R_{H}$ should be universal according to the standard renormalization group (RG) arguments. It is quite analogous to the amplitude ratio of the magnetic susceptibility of a spin system near critical temperature (see, for instance, [130]). It is known that the ratio of the amplitude of the magnetic susceptibility in the high-temperature phase to that of the low-temperature phase is universal in many spin systems near critical temperatures. We therefore suggest that the ratio $R_{G}/R_{H}$ of a given topological polymer should be almost the same for different models of topological polymers.

In order to confirm how universal the ratio $R_{G}/R_{H}$ is, we analytically show that the ratio of the gyration radius to the hydrodynamic radius of a topological polymer, $R_{G}/R_{H}$, is characterized by the variance of the probability distribution function of the distance between two segments of the polymer (see Section 3.3). We argue that the ratio decreases if the variance decreases. We call this function the distance distribution function. It is expressed with the pair distribution function, or the radial distribution function of polymer segments [133].

We numerically evaluate the distance distribution function of a topological polymer both for the ideal and real chain models. In the case of ideal ring polymers, we exactly derive an analytic expression of the pair distribution function (see Section 4.2).

For real topological polymers, we show that the short-distance intrachain correlation is much enhanced. The correlation hole becomes large, if the graph is complex (see Section 4.4). The exponent of the short-range power-law behavior in the distance distribution function is given by 0.7 for a linear polymer and a ring polymer, while it is given by larger values such as 0.9 and 1.15 for a θ-shaped polymer and a complete bipartite graph $K_{3,3}$ polymer, respectively, as shown in [31].

We argue that the estimate to the exponent of the short-distance correlation such as 0.7 for linear and ring chains should be consistent with the estimate of the exponent $\theta_{2}$ of the short-range correlation in a self-avoiding walk (SAW) derived by des Cloizeaux through the RG arguments [134]. Here, we remark that the estimate of $\theta_{2}$ has been confirmed in the simulation of SAW [135].

### 1.4. Contents of the Review

Let us briefly explain the motivations for the present review. We believe that the field of topological polymers is still progressing rapidly and further growing currently. However, there are already several different approaches on the subject such as the linear time algorithm based on the quaternions for generating such random walks connecting two given points, the Kremer–Grest model of molecular dynamics, and so on. If we regard knotted ring polymers as a subclass of topological polymers, there are already several results on them. Furthermore, it should be interesting to interpret some results from the RG viewpoint such as for the ratio of the gyration radius to the hydrodynamic radius in terms of the variance to the mean-square distance among segments of a given polymer. Incidentally, the viewpoint of semi-flexibility is quite suitable for the Special Issue of Polymers. In fact, as emphasized in Section 1.1, DNA chains are thin and stiff, so that they are easily knotted. Probably, this is the reason why knotted ring polymers were synthesized in the experiment of circular DNA in the 1980s. Thus, we consider that it is time to make a concise review of the currently available approaches and results of topological polymers.

The contents of the present review consist of the following. In Section 2, we refer to some relevant papers on the quaternion algorithm for generating topological polymers with a given graph and then briefly explain the time evolution of the Kremer–Grest model in molecular dynamics. In Section 3, we present the data of the mean-square radius of gyration and the hydrodynamic radius both for ideal and real topological polymers. We argue that the ratio of the root mean-square radius of gyration $R_{G}$ to the hydrodynamic radius $R_{H}$ for a topological polymer decreases if the variance of the distance distribution function decreases. In Section 4, we evaluate the distance distribution functions both for ideal and real topological polymers. For ideal ones, they are consistent with the exact result for ideal ring polymers. For real topological polymers, we numerically show that short-range intrachain correlation is much enhanced, i.e., the exponent of the short-range behavior in the distance distribution function becomes large, for topological polymers with complex graphs. In Section 5, we introduce the knotting probability, which corresponds to the reduced entropy of a ring polymer under a given topological constraint. In Section 6, we review theoretical studies on the average size of a knotted ring polymer in solution. We show that the mean square radius of gyration of a ring polymer under a topological constraint can be larger than that of no topological constraint if the excluded volume is small.

## 2. Topological Polymers with Various Graphs and Numerical Methods

### 2.1. Numerical Method I: Ideal Topological Polymers

#### 2.1.1. Linear Time Algorithm Based on Quaternions

By the method of quaternions, we can generate a large number of random conformations of an ideal topological polymer expressed by a graph quite fast [31,126,127]. It gives a linear time algorithm: the computation time is proportional to the number of segments of a given topological polymer. The sampling method for generating random walks with a given end-to-end distance has been shown [31,127].

Let us review briefly how the linear time method for generating random configurations of topological polymers with a given graph has been developed. Hausmann and Knutson introduced a useful description of closed *n*-edge three-dimensional polygons of total length two [136]. They were interested in it primarily as a way to analyze the symplectic and algebraic geometry of polygon space (see also [137,138]). Through the map a probability measure on closed three-dimensional polygons has been introduced in [126], by which we can construct an ensemble of configurations of ideal topological polymers with any given graph *F* [31,127]. The analysis of polygon space through symplectic geometry is further extended [139], and a fast algorithm for constructing equilateral random polygons has been introduced [140].

We give three remarks. (i) The mathematical proof of the sampling method for generating random walks with a given end-to-end distance will be shown in [141]. (ii) The quaternion method for generating ideal topological polymers is practically useful for evaluating physical quantities. In fact, we can estimate at least approximately the values of $R_{G}^{2}$ or $R_{H}$ for real topological polymers by making use of the ratios among the corresponding ideal ones, if the valency of vertices is limited up to three. Moreover, the ratio of the gyration radius to the hydrodynamic radius of a topological polymer, $R_{G}/R_{H}$, is characteristic of the structure of chemical connectivity expressed by the graph of the polymer. (iii) There are other methods for generating closed equilateral random walks, i.e., equilateral random polygons. For instance, they are generated incrementally step-by-step in [142,143].

#### 2.1.2. Generating Random Configurations of Ideal Topological Polymers

We construct weighted ensembles of random configurations for the topological polymer of a graph and numerically evaluate the expectation value of a physical quantity by taking the weighted sum for the quantity [31,127,141].

We first generate random configurations for the open sub-chains and closed sub-chains, which are part of the given graph, and we attach appropriate weights to the open sub-chains of the graph. We determine the weight of the configuration for the whole graph by taking the product of all of the weights assigned to the open sub-chains of the graph.

Theta curve graph (θ-shaped graph):

We generate weighted random configurations of a theta-curve graph polymer of N=3n segments as follows [127]. We first construct random polygons of 2n segments by the method of quaternions. Second, we take two antipodal points A and B on the polygon such that each of the sub-chains connecting A and B has *n* segments. Third, we connect the points A and B by an *n*-step random walk with its end-to-end distance equal to the distance between the points A and B by making use of the method of quaternions.

To the whole configuration we assign the weight $w_{\mathrm{AB}}$, which is given by the *N*-step Gaussian probability density of the end-to-end vector between the points A and B.

### 2.2. Numerical Method II: Molecular Dynamics Simulation

#### The Kremer–Grest Model through LAMMPS

A polymer chain in the Kremer–Grest model has both the repulsive Lennard–Jones (LJ) potentials and the finitely extensible nonlinear elongation (FENE) potentials to prevent the bonds from crossing each other [128]. We introduce the cutoff in order to produce a repulsive Lennard–Jones potential.

We generate an ensemble of conformations of a real topological polymer of graph *F* for the Kremer–Grest model by LAMMPS [144]: the initial conformation is given by putting the atoms on the lattice points along the polygonal lines of the given graph *F* in a cubic lattice. We performed the MD simulation in NVEensembles with Langevin thermostat (i.e., NVE and Langevin fix in LAMMPS). Then, by LAMMPS, we integrate Newton’s equation of motion for the atoms under the repulsive Lennard–Jones and FENE potentials [145].

The topological type of the conformation of a real topological polymer in the Kremer–Grest model does not change during time evolution. The bonds can hardly cross each other, since the atoms are surrounded by strong barriers, which increase with respect to the inverse of $r_{ij}^{12}$ while they are connected with nonlinear elastic springs of finite length (see also [103]).

## 3. Gyration and Hydrodynamic Radii of Topological Polymers

### 3.1. Mean-Square Radius of Gyration for Topological Polymers

We define the mean-square radius of gyration for a topological polymer of graph *F* consisting of *N* segments by:

(1) $${\langle R_{G}^{2}\rangle }_{F}=\frac{1}{N}\sum_{j=1}^{N}{\langle {\left({\vec{r}}_{j}-{\vec{R}}_{c}\right)}^{2}\rangle }_{F}\;.$$

Here, the symbol ${\langle A\rangle }_{F}$ denotes the ensemble average of quantity *A* over all possible configurations of the topological polymer with graph *F*, and ${\vec{R}}_{c}$ the position vector of the center of mass. We remark that ${\vec{r}}_{j}$ denote the position vectors of the *j*-th segments for j=1,2,…,N.

We denote by $R_{G}\left(F\right)$ the square root of the mean-square radius of gyration ${\langle R_{G}^{2}\rangle }_{F}$:

(2) $$R_{G}\left(F\right)=\sqrt{{\langle R_{G}^{2}\rangle }_{F}}\;.$$

We also call it the gyration radius of the polymer.

**Figure 3:**
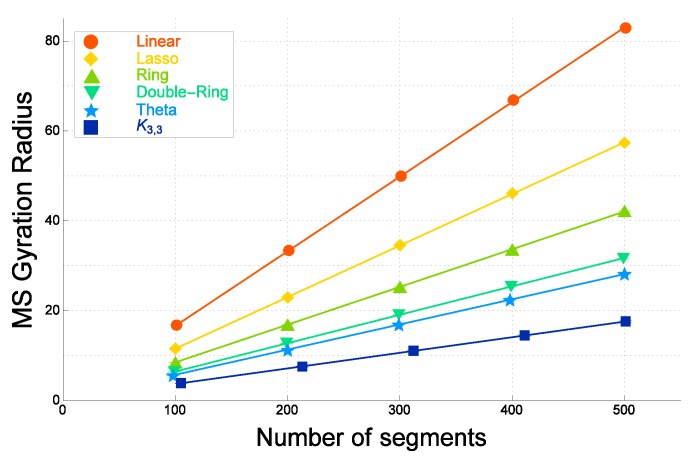
Mean-square radius of gyration ${\langle R_{G}^{2}\rangle }_{F}$ versus the number of segments *N* for ideal topological polymers with graph *F* evaluated by the quaternion method for six graphs *F*, such as linear, tadpole (lasso), ring, double-ring, θ-shaped and complete bipartite graph $K_{3,3}$ polymers, depicted by filled circles, filled diamonds, filled upper triangles, filled lower triangles, filled stars and filled squares, respectively. Each data point corresponds to the average over $10^{4}$ samples. The error bars are too small to be seen. Reprinted with permission from [31].

In Figure 3, the numerical estimates of the mean-square radius of gyration ${\langle R_{G}^{2}\rangle }_{F}$ for ideal topological polymers of graph *F* are plotted against the number of segments *N* for six different graphs. They are evaluated by the quaternion method [31]. They are given in decreasing order for each given number of segments *N* as follows: those of linear polymers, tadpole (lasso) polymers, ring polymers, double-ring polymers, θ-shaped polymers and polymers with a complete bipartite graph $K_{3,3}$.

As shown in Figure 3, the estimate of the gyration radius for a double-ring polymer of *N* segments, depicted by filled lower triangles, is close to that of a θ-shaped polymer of *N* segments depicted with filled stars. The former is only slightly larger than the latter. For various other graphs, the estimates of the mean-square radius of gyration are distinct among the different graphs with the same given number of segments *N*.

The estimates of the mean-square radius of gyration for the ideal topological polymers linearly depend on the number of segments *N*, as shown in Figure 3. They are fitted by the formula:

(3) $${\langle R_{G}^{2}\rangle }_{F}=a_{0}+a_{1}N\;.$$

The best estimates of parameters $a_{0}$ and $a_{1}$ in (3) are given in [31]. The $\chi^{2}$ values per degrees of freedom (DF) for the topological polymers with the different graphs are at most 1.1 and are small. Here, we remark that the absolute values of $a_{0}$ are less than 0.2 and small.

For real topological polymers, which have excluded volume, the numerical values of the mean-square radius of gyration are proportional to some power of the number of segments *N* with scaling exponent ν=0.59, if the number of segments *N* is large enough. We have good theoretical curves fitted to the data by applying the following formula:

(4) $${\langle R_{G}^{2}\rangle }_{F}=a_{0}+a_{1}\;N^{2\nu }\;.$$

Here, we fix the value of scaling exponent ν by ν=0.59 when we apply Formula (4) to the data in order to derive the best estimates of parameters $a_{0}$ and $a_{1}$.

**Figure 4:**
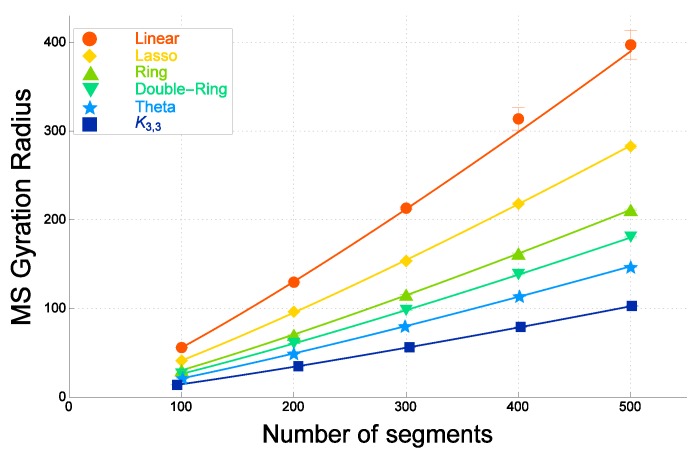
Mean-square radius of gyration ${\langle R_{G}^{2}\rangle }_{F}$ versus the number of segments *N* for real topological polymers with graphs *F* evaluated by the molecular dynamics of the Kremer–Grest model. Each data point corresponds to the average over $5\times 10^{3}$ samples. Reprinted with permission from [31].

In Figure 4, the estimate of the gyration radius for a double-ring polymer of *N* segments is clearly larger than that of a θ-shaped polymer of *N* segments, for each *N*. The former and the latter are distinct for each segment number *N*. This is in agreement with Tezuka’s observation in experiments that if a polymer has such a vertex with four connecting bonds, the average size of the polymer is much enhanced due to the excluded volume effect [129]. Here, we remark that a double-ring polymer indeed has a vertex where four bonds are connected. We recall that in the case of ideal topological polymers, the size of double-ring polymers is close to that of θ-shaped polymers with the same number of segments *N*.

It has been shown in the MC simulation [28] that in the case of nonzero finite excluded volume, the mean-square radius of gyration for double-ring polymers of *N* segments is much closer to that of ring polymers of *N* segments, while in the case of no excluded volume, the mean-square radius of gyration for double-ring polymers of *N* segments is distinctly smaller than that of ring polymers of *N* segments.

We now argue that the ratios among the estimates of the mean-square radius of gyration for ideal topological polymers with graphs *F* are approximately similar to those of real topological polymers with the same such graphs *F* consisting of at most trivalent vertices, as far as we have investigated.

Let us first consider the estimates of coefficient $a_{1}\left(F\right)$ in Formula (3) for ideal topological polymers with graphs *F*. If *N* is large enough, the coefficients $a_{1}$ determine the ratios among the gyration radii $R_{G}\left(F\right)$ for some graphs *F*. We thus calculate the ratio $a_{1}\left(F\right)/a_{1}\left(\mathrm{ring}\right)$ for ideal topological polymers of graph *F* in order to study the ratios among the gyration radii of topological polymers with different graphs. They are listed in Table 1. We then consider the estimates of coefficient $a_{1}\left(F\right)$ in Formula (4) for real topological polymers with graphs *F*. We again calculate the ratios $a_{1}\left(F\right)/a_{1}\left(\mathrm{ring}\right)$ for those of real topological polymers. They are listed in the third column of Table 1.

For ideal polymers, the ratio $a_{1}\left(\mathrm{linear}\right)/a_{1}\left(\mathrm{ring}\right)$ is given by 1.98, which is almost equal to 2.0 with respect to errors, while for real polymers, it is given by a smaller value of 1.84. It is clearly smaller than 2.0 with respect to errors. Thus, the ratio $a_{1}\left(\mathrm{linear}\right)/a_{1}\left(\mathrm{ring}\right)$ is smaller than 2.0 for real polymers.

We suggest that for the real linear and ring polymers, the ratio $a_{1}\left(\mathrm{linear}\right)/a_{1}\left(\mathrm{ring}\right)$ is smaller than 2.0 due to the excluded volume effect. In fact, the mean-square radius of gyration is enhanced through the effective repulsions among segments under the excluded volume. We therefore conclude that the excluded volume is more important in ring polymers than in linear polymers.

The excluded volume plays a significant role in a double-ring polymer, for which one vertex has four valencies [28]. In fact, the ratio $a_{1}\left(\mathrm{double}\;\;\mathrm{ring}\right)/a_{1}\left(\mathrm{ring}\right)$ for a real double-ring polymer is distinctly larger than that of an ideal double-ring polymer. In Table 1, the former is given by 0.85, while for the latter given by 0.75. The former is distinctly larger than the latter with respect to errors.

### 3.2. Diffusion Coefficient via the Hydrodynamic Radius for a Topological Polymer

We evaluate the diffusion coefficients of topological polymers in solution by applying Kirkwood’s approximation [131,132,133]. The diffusion coefficient, D(F), of a topological polymer with graph *F* consisting of *N* segments in a solvent with viscosity η at a temperature *T* is given by:

(5) $$D\left(F\right)=\frac{k_{B}T}{6\pi \eta }\frac{1}{N^{2}}\sum_{n,m=1;n\neq m}^{N}\langle \frac{1}{|{\vec{r}}_{m}-{\vec{r}}_{n}|}\rangle_{F}\;.$$

Here, we take the average of the inverse distance between two segments over all pairs of segments of a given topological polymer. We introduce the hydrodynamic radius $R_{H}\left(F\right)$ of a topological polymer with graph *F* of *N* segments by:

(6) $$\frac{1}{R_{H}\left(F\right)}=\frac{1}{N^{2}}\sum_{n,m=1;n\neq m}^{N}\langle \frac{1}{|{\vec{r}}_{m}-{\vec{r}}_{n}|}\rangle_{F}\;.$$

**Figure 5:**
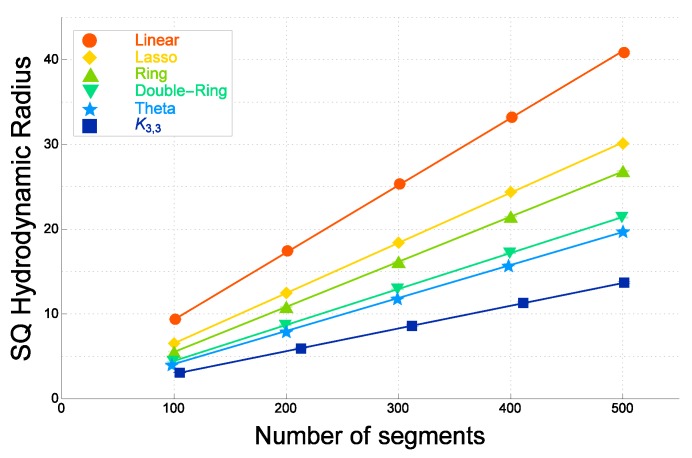
Hydrodynamic radius squared ${\left(R_{H}\left(F\right)\right)}^{2}$ versus the number of segments *N* for ideal topological polymers of graphs *F* evaluated by the quaternion method. The error bars are too small to be seen. Reprinted with permission from [31].

In Figure 5, we plot against the number of segments *N* the square of the hydrodynamic radius, ${\left(R_{H}\left(F\right)\right)}^{2}$, for ideal topological polymers with graphs *F* evaluated by the quaternion method.

The curves fitted to the data points are given by applying the following formula:

(7) $${\left(R_{H}\left(F\right)\right)}^{2}=a_{0}+a_{1}N\;.$$

We observe in Figure 5 that the *N*-dependence of the hydrodynamic radius is well approximated by the fitted curves of Formula (7). However, the curves are not very good since the $\chi^{2}$/DF are not very small. In fact, if we apply a simple power of *N* to the data such as $N^{2\nu }$, the estimates of scaling exponent ν are less than 0.5 [31]. In experiments, the data points of the hydrodynamic radius versus the number of segments for linear polymers in a θ solvent are fitted by a curve with exponent ν=0.484 [133,146].

In Figure 5, the hydrodynamic radius $R_{H}\left(F\right)$ of an ideal double-ring polymer of *N* segments is close to, but slightly larger than that of an ideal θ-shaped polymer of *N* segments. It is similar to the case of the mean-square radius of gyration.

**Figure 6:**
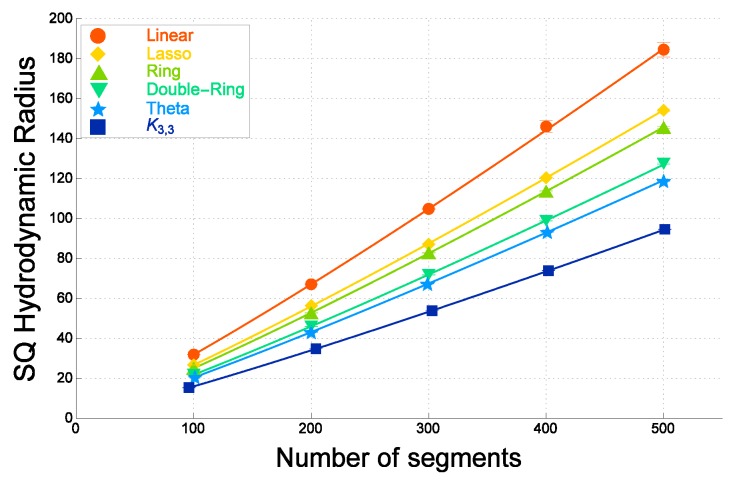
Hydrodynamic radius squared ${\left(R_{H}\left(F\right)\right)}^{2}$ versus the number of segments *N* for real topological polymers of graphs *F* (the Kremer–Grest model). The error bars are too small to be seen. Reprinted with permission from [31].

In Figure 6, we plot against the number of segments *N* the hydrodynamic radius for real topological polymers evaluated by performing the molecular dynamical simulation of the Kremer–Grest model through LAMMPS. Here, we recall that each molecule has excluded volume in the model.

The fitted curves are given by applying the following formula to the data points:

(8) $${\left(R_{H}\left(F\right)\right)}^{2}=a_{0}+a_{1}N^{1.13}\;.$$

It corresponds to Formula (4) in which we put ν=0.565. The best estimates of the parameters $a_{0}$ and $a_{1}$ are give in [31]. Here, the value of the exponent ν=0.565 is slightly smaller than the scaling exponent of SAW: $\nu_{\mathrm{SAW}}=0.588$. However, it is also the case in polymer experiments such as ν=0.567 [133,147].

We observe in Figure 6 that the hydrodynamic radius $R_{H}$ of real double-ring polymers is approximately close to, but distinctly larger than that of real θ-shaped polymers. The enhancement due to the excluded volume is less significant than in the case of the mean-square radius of gyration in the range from N=100 to 500. The ratio of $a_{1}$ for double-ring and θ-shaped polymers in the case of real polymers is smaller than that of ideal polymers.

### 3.3. Ratio $R_{G}/R_{H}$ in Terms of the Mean-Square Deviations of the Distance between Two Segments: Universality of the Ratio

We now consider the ratio of the gyration radius $R_{G}$ to the hydrodynamic radius $R_{H}$ for topological polymers whose structures in chemical connectivity are expressed by graphs *F*. We suggest that the ratio is universal in the sense of the renormalization group arguments, i.e., we expect that it is independent of some details of the polymer model, so that we can compare the estimate of the ratio with other theoretical approaches and experiments. Here, we take the analogy of the amplitude ratio of susceptibilities and that of specific heats in critical phenomena [130].

We now argue that the ratio of the gyration radius to the hydrodynamic radius, $R_{G}/R_{H}$, decreases if the standard deviation σ becomes small compared with the mean distance $\bar{r}$ for the distance *r* between a randomly chosen pair of segments of a polymer [31]. Here, we denote by $\bar{r}$ and $\sigma^{2}$ the mean value and the variance of the distance *r* between an arbitrary pair of segments, respectively. The arguments also suggest that the ratio $R_{G}/R_{H}$ should be universal.

We first recall that the mean-square radius of gyration of a polymer is given by the average of the square distance between two segments over all of the pairs:

(9) $$R_{G}^{2}=\frac{1}{2N^{2}}\sum_{j,k=1}^{N}\langle {\left({\vec{r}}_{j}-{\vec{r}}_{k}\right)}^{2}\rangle \;.$$

It is therefore expressed in terms of the mean-square distance between two segments of the polymer, $\langle r^{2}\rangle$, as follows.

(10) $$R_{G}^{2}=\langle r^{2}\rangle /2\;.$$

Through Kirkwood’s approximation, the hydrodynamic radius is given by the average of the inverse distance between two segments over all of the pairs of segments:

(11) $$R_{H}^{-1}=\langle 1/r\rangle \;.$$

Thus, the gyration radius is given by the root of the mean-square of the distance between two segments, while the hydrodynamic radius by its harmonic mean.

Let us express the distance *r* between an arbitrary pair of segments in a polymer in terms of the mean distance $\bar{r}$ and the deviation Δr as $r=\bar{r}+\Delta r$. The variance $\sigma^{2}$ of the distance *r* is given by:

(12) $$\sigma^{2}=\langle {\left(\Delta r\right)}^{2}\rangle \;.$$

The mean-square of the distance *r* is therefore given by:

(13) $$\begin{array}{ccc} \langle r^{2}\rangle & = & \langle {\left(\bar{r}+\Delta r\right)}^{2}\rangle \\ & = & {\bar{r}}^{2}+\sigma^{2}\;. \end{array}$$

If the mean distance $\bar{r}$ is much larger than the standard deviation σ, we may assume that the inverse distance is expressed as an infinite series of Δr: $1/r=1/\bar{r}-\Delta r/{\bar{r}}^{2}+\cdots$.

We thus have the following approximation:

(14) $$\langle 1/r\rangle \approx 1/\bar{r}+\sigma^{2}/{\bar{r}}^{3}\;.$$

The ratio of the gyration radius to the hydrodynamic radius is therefore approximately given by:

(15) $$R_{G}/R_{H}\approx \frac{1}{\sqrt{2}}\;{\left(1+\frac{\sigma^{2}}{{\bar{r}}^{2}}\right)}^{3/2}\;.$$

We have thus shown that the ratio $R_{G}/R_{H}$ of a given polymer mainly depends on the ratio of the variance to the mean-square distance among segments of the polymer. The result of Equation (15) supports the universality of the ratio $R_{G}/R_{H}$. For the two different models of topological polymers given, although the distance distribution functions are different, if they have the same ratio of the variance to the mean-square distance, the ratio $R_{G}/R_{H}$ becomes the same for them.

### 3.4. Estimates of the Ratio of the Gyration to Hydrodynamic Radii

**Figure 7:**
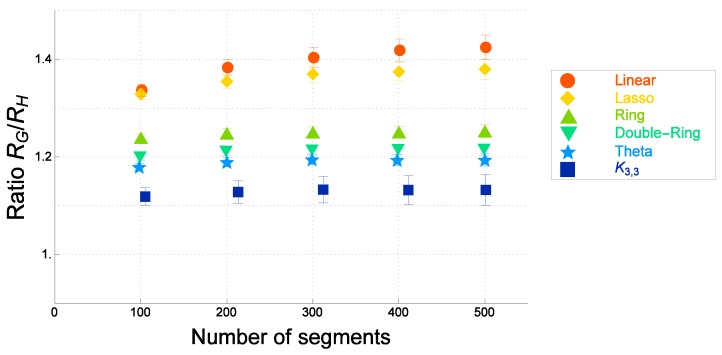
Ratio of the gyration to hydrodynamic radii, $R_{G}\left(F\right)/R_{H}\left(F\right)$, versus the number of segments *N* for ideal topological polymers with graphs *F* evaluated by the quaternion method. Reprinted with permission from [31].

**Figure 8:**
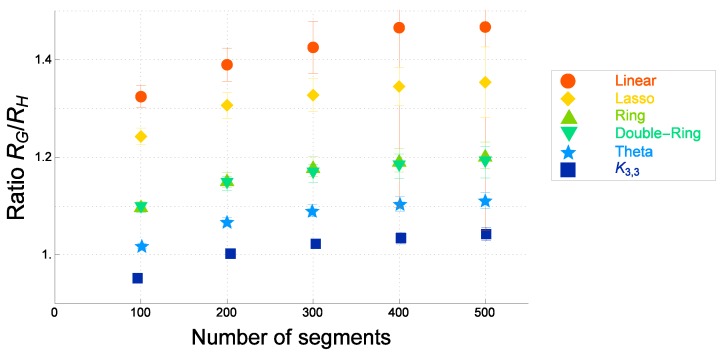
Ratio of the gyration to hydrodynamic radii $R_{G}\left(F\right)/R_{H}\left(F\right)$ versus the number of segments *N* for real topological polymers (the Kremer–Grest model). Reprinted with permission from [31].

We numerically evaluate the ratio $R_{G}/R_{H}$ for several topological polymers with various different graphs *F*. For ideal topological polymers of graphs *F*, the numerical estimates of the ratio $R_{G}/R_{H}$ are plotted against the number of segments *N* in Figure 7. For real topological polymers with graph *F* (the Kremer–Grest model), the estimates of the ratio of the gyration radius to the hydrodynamic radius, $R_{G}\left(F\right)/R_{H}\left(F\right)$, are plotted against the number of segments *N* in Figure 8.

By comparing Figure 7 and Figure 8, we observe that the estimates of the ratio of the gyration radius to the hydrodynamic radius are given in the same order for both the ideal and the real topological polymers of graphs *F*. The ratios are given in decreasing order by that of a linear polymer, tadpole (lasso) polymer, ring polymer, double-ring polymer, θ-shaped polymer and the polymer of a complete bipartite graph $K_{3,3}$, for ideal topological polymers, as shown in Figure 7. It is also the case for real topological polymers, as shown in Figure 8.

In each of Figure 7 and Figure 8, the ratio $R_{G}\left(F\right)/R_{H}\left(F\right)$ increases with respect to the number of segments *N*. As the number of segments *N* becomes very large such as N=500, the ratios approach constant values for all graphs *F* as shown in Figure 7 and Figure 8. The ratios among topological polymers with the graphs *F* are given by almost the same values both for the ideal topological polymers and the real topological polymers.

The ratios at N=500 for topological polymers with various graphs *F* are listed in Table 2. In each case of graphs *F*, the ratio for the ideal topological polymer and that of the real topological polymer is given by almost the same values with respect to errors. We thus suggest that the estimates of the ratio $R_{G}\left(F\right)/R_{H}\left(F\right)$ should be universal if the number of segments *N* is large enough such as N≥500.

## 4. Correlation Functions of Topological Polymers

### 4.1. Exponents of Short Distance Intrachain Correlation of SAW

We now review that the short-distance intrachain correlation of a polymer chain is nontrivial even for linear polymers and also that it is closely related to the probability distribution function of the distance between a given pair of segments.

In order to describe the intrachain correlations of a SAW [130,148], we denote by $p_{0}\left(r\right)$ the probability distribution function of the end-to-end distance *r* of an *N*-step SAW. For the short-distance behavior [149,150], we have:

(16) $$p_{0}\left(r\right)∼y^{g}\;\left(r<R_{N}\right).$$

Here, $R_{N}=R_{0}N^{\nu }$ with some constant $R_{0}$, and the exponent *g* is given by g=(γ+1−α−dν)/ν through asymptotic analysis [150] and g=(γ−1)/ν through the RG arguments [151] (for definitions of critical exponents, see [130]). It was shown that the large-distance asymptotic behavior of $p_{0}\left(r\right)$ for $r>R_{N}$ is given by [149,150]:

(17) $$p_{0}\left(r\right)∼R_{N}^{-d}A\left(r/R_{N}\right)\exp\left(-{\left(r/R_{N}\right)}^{\delta }\right)\;,$$

where A(y) does not change exponentially fast for large *y*. Here, exponent δ is given in terms of exponent ν as δ=1/(1−ν).

We remark that the asymptotic properties of $p_{0}\left(r\right)$ are studied theoretically [152,153,154] and numerically [155,156,157,158,159,160].

In order to study the short-distance intrachain correlation between two segments of a long polymer in a good solvent, let us denote by $p_{1}\left(r\right)$ and $p_{2}\left(r\right)$ the probability distribution function between a middle point and an end point of an *N*-step SAW and that of two middle points, respectively. Then, we define critical exponents $\theta_{s}$ for s=0,1,2 as follows. Assuming $p_{s}\left(r\right)=R_{N}^{-d}F_{s}\left(r/R_{N}\right)$, we have:

(18) $$F_{s}\left(y\right)∼y^{\theta_{s}}\;\left(r<R_{N}\right)\;\mathrm{for}\;s=0,1,2.$$

The RG estimates of the exponents for d=3 are given by $\theta_{0}=0.273,\theta_{1}=0.46$ and $\theta_{2}=0.71$, respectively [134]. They are close to the MC estimates, such as $\theta_{0}=0.23\pm 0.02,\theta_{1}=0.35\pm 0.03$ and $\theta_{2}=0.74\pm 0.03$, respectively [135].

The RG and MC estimates of exponent $\theta_{2}$ are in agreement with the value 0.7 of the exponent for the short-range power-law behavior of the distance distribution functions of real linear and real ring polymers within errors. Here, we remark that most of the pairs of segments in a SAW are given by those between middle points.

### 4.2. Exact Expression of the Pair Distribution Function of a Gaussian Ring Polymer

Let us define the local segment density $\rho (\vec{r})$ in terms of the position vectors ${\vec{r}}_{j}$ of the *j*-th segments of a polymer consisting of *N* segments for j=1,2,…,N, by:

(19) $$\rho \left(\vec{r}\right)=\sum_{j=1}^{N}\delta \left(\vec{r}-{\vec{r}}_{j}\right)\;.$$

We call $\langle \rho \left({\vec{r}}_{1}\right)\rho \left({\vec{r}}_{2}\right)\rangle$, the statistical average of the product of the local densities at ${\vec{r}}_{1}$ and ${\vec{r}}_{2}$, the pair distribution function [133]. Since the polymer system is homogeneous and isotropic in the ensemble average, we denote the pair distribution function $\langle \rho \left(\vec{r}\right)\rho \left(\vec{0}\right)\rangle$ simply by 〈ρ(r)ρ(0)〉 with $r=|\vec{r}|$. It is related to the radial distribution function of polymer segments, g(r), as $g\left(r\right)=\langle \rho \left(r\right)\rho \left(0\right)\rangle /\rho^{2}$.

We can show that the average number of segments per volume located at $\vec{r}$ when there is already a segment at the origin $\vec{r}=\vec{0}$ is given by [133]:

(20) $$\langle \rho \left(\vec{r}\right)\rho \left(\vec{0}\right)\rangle /\rho \;.$$

Let us consider a Gaussian linear chain of *N* steps with bond length *b*. For a given ${\vec{r}}_{2}$, the probability density for ${\vec{r}}_{1}$ is given by:

(21) $$G\left({\vec{r}}_{1},{\vec{r}}_{2};N\right)={\left(2\pi Nb^{2}/3\right)}^{-3/2}\exp\left(-\frac{{\left({\vec{r}}_{1}-{\vec{r}}_{2}\right)}^{2}}{2Nb^{2}/3}\right)\;.$$

For a Gaussian linear chain of *N* steps, the pair distribution function is given by [133]:

(22) $$\begin{array}{c} \langle \rho \left(\vec{r}\right)\rho \left(0\right)\rangle /\rho =\frac{1}{N}\int_{0}^{N}dn\int_{0}^{N}G(\vec{r},\vec{0};|n-n^{{}^{\prime }}\left|\right)dn^{{}^{\prime }}\;\\ =\frac{6}{\pi^{3/2}b^{2}r}\int_{\sqrt{u}}^{\infty }\left(1-u/x^{2}\right)\exp\left(-x^{2}\right)dx \end{array}$$

where *u* is given by $u=3r^{2}/\left(2Nb^{2}\right)$. For small *r*, we have:

(23) 〈ρ(r)ρ(0)〉/ρ≈1/r

and for large *r*, we have with $R_{G}^{2}=Nb^{2}/6$:

(24) $$\langle \rho \left(r\right)\rho \left(0\right)\rangle /\rho \approx \frac{1}{r^{4}}\exp\left(-r^{2}/4R_{G}^{2}\right)\;.$$

For the Gaussian ring chain of *N* steps, we express the pair distribution function in terms of the probability densities of the two random walks with *n* and N−n steps connecting the origin and $\vec{r}$ as follows.

(25) $$\begin{array}{c} \langle \rho \left(\vec{r}\right)\rho \left(\vec{0}\right)\rangle /\rho =\frac{1}{N}\int_{0}^{N}dn\int_{0}^{N}dn^{{}^{\prime }}G(\vec{r},\vec{0};|n-n^{{}^{\prime }}|)G(\vec{0},\vec{r};N-|n-n^{{}^{\prime }}\left|\right)\;\\ \;\times {\left(G(\vec{0},\vec{0};N)\right)}^{-1}\;. \end{array}$$

Here, we remark that the normalization factor in (25) is derived as follows.

(26) $$G\left(\vec{0},\vec{0};N\right)=\int_{V}d^{3}\vec{r}\;\left(\frac{1}{N}\int_{0}^{N}dnG\left(\vec{0},\vec{r};n\right)G\left(\vec{r},\vec{0};N-n\right)\right)\;.$$

We can show that Equation (25) is expressed as:

(27) $$\langle \rho \left(\vec{r}\right)\rho \left(\vec{0}\right)\rangle /\rho =\int_{0}^{N}G\left(\vec{r},\vec{0};n\right)G\left(\vec{0},\vec{r};N-n\right)dn/G\left(\vec{0},\vec{0};N\right)\;.$$

By calculating the integral (27) with respect to variable *n*, we derive exactly an explicit expression of the pair distribution function of the Gaussian polygon of *N* steps:

(28) $$\langle \rho \left(r\right)\rho \left(0\right)\rangle /\rho =\frac{N}{2\pi v^{2}}\;\frac{1}{r}\exp\left(-\frac{r^{2}}{v^{2}}\right)\;,$$

where $v^{2}$ is given by:

(29) $$v^{2}=Nb^{2}/6\;.$$

Here, we recall that for random polygons, we have $R_{G}^{2}=Nb^{2}/12$, and hence, we have $v^{2}=2R_{G}^{2}$. The derivation of (28) is given in Appendix B of [31].

The exact expression (28) of the pair distribution function leads to the integral expression of the static structure factor derived by Casassa [11].

### 4.3. Gyration and Hydrodynamic Radii in Terms of the Distance Distribution Function

We denote by F(r) the probability distribution function of the distance *r* between a given pair of segments of a polymer. That is, F(r)dr gives the probability that the distance between two given segments is larger than *r* and smaller than r+dr. We also call it the distance distribution function. It is related to the pair distribution function as:

(30) $$N\;F\left(r\right)\;dr=\frac{1}{\rho }\langle \rho \left(r\right)\rho \left(0\right)\rangle \;4\pi r^{2}dr\;.$$

Let us denote by $r_{\mathrm{RMS}}$ the square root of the mean-square distance between two segments of a polymer. We define it by:

(31) $${\left(r_{\mathrm{RMS}}\right)}^{2}=\int_{0}^{\infty }r^{2}\;F\left(r\right)\;dr\;.$$

In terms of $r_{\mathrm{RMS}}$, we define the normalized distance by:

(32) $$x=r/r_{\mathrm{RMS}}.$$

We then define the probability distribution function f(x) of the normalized distance *x* by:

(33) $$F\left(r\right)\;dr=f\left(r/r_{\mathrm{RMS}}\right)\;dr/r_{\mathrm{RMS}}\;.$$

That is, we have assumed F(r)dr=f(x)dx. We also call f(x) the normalized distance distribution, briefly.

It follows from (31) that not only the zeroth moment, but also the second moment of the normalized distance distribution is given by one. The mean-square radius of gyration is expressed as:

(34) $$\begin{array}{ccc} R_{G}^{2} & = & \frac{1}{2}\;\int_{0}^{\infty }r^{2}\;F\left(r\right)\;dr\;. \end{array}$$

We have:

(35) $$R_{G}^{2}=r_{\mathrm{RMS}}^{2}/2\;,$$

and $R_{G}=r_{\mathrm{RMS}}/\sqrt{2}$. The hydrodynamic radius is expressed in terms of the distance distribution function as:

(36) $$\begin{array}{ll} \frac{1}{R_{H}} & =\int_{0}^{\infty }\frac{1}{r}\;F\left(r\right)\;dr\\ & =\frac{1}{r_{\mathrm{RMS}}}\;\int_{0}^{\infty }x^{-1}f\left(x\right)dx. \end{array}$$

The hydrodynamic radius $R_{H}$ is given by the harmonic mean of the distance between two segments of the polymer. We therefore have the ratio of the gyration radius to the hydrodynamic radius as:

(37) $$R_{G}/R_{H}=\frac{1}{\sqrt{2}}\;\int_{0}^{\infty }x^{-1}f\left(x\right)dx\;.$$

**Figure 9:**
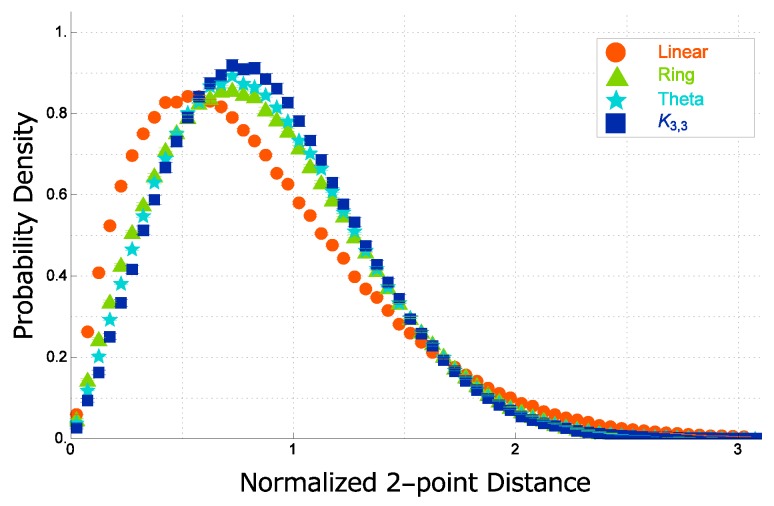
Probability distribution function f(x) of the normalized distance *x* between any given pair of segments in an ideal topological polymer of N=500 with graph *F*, where the distance between two segments is normalized by the square root of the mean-square distance $r_{\mathrm{RMS}}$ of the topological polymer with graph *F*. Here, graph *F* is given by a linear chain (red circles), a ring chain (green upper triangles), a theta curve (light blue stars) and a complete bipartite graph $K_{3,3}$ (dark blue squares). Reprinted with permission from [31].

In Figure 9, the data points of the normalized distance distribution f(x) are plotted against the normalized distance $x=r/r_{\mathrm{RMS}}$. The three curves are almost overlapping and only slightly different from each other. However, such small difference may lead to quite distinct estimates for the ratio of the gyration radius to the hydrodynamic radius, $R_{G}/R_{H}$.

The normalized distance distribution f(x) of an ideal topological polymer with graph *F* is well approximated by the following:

(38) $$f\left(x\right)=Cx^{\theta }\exp\left(-x^{\delta }\right)\;.$$

Substituting (38) to the integral of (37), we have:

(39) $$R_{G}/R_{H}=\frac{1}{\sqrt{2}}\frac{C}{\delta }\Gamma \left(\theta /\delta \right)\;.$$

For the Gaussian ring polymer, the ratio $R_{G}/R_{H}$ is exactly given by:

(40) $$R_{G}/R_{H}=\sqrt{\frac{\pi }{2}}\;.$$

The numerical value $\sqrt{\pi /2}\approx 1.253314$ is rather close to the estimate obtained by the quaternion method presented in Table 2.

### 4.4. Real Topological Polymers Showing Enhanced Short-Range Correlation

**Figure 10:**
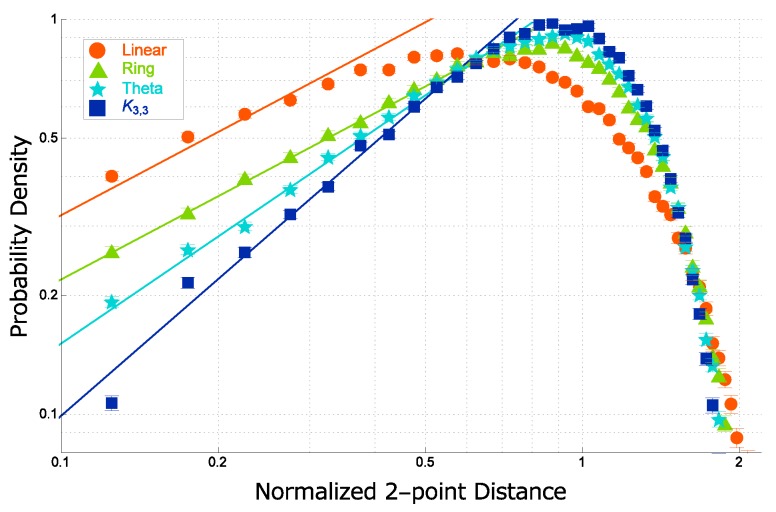
Double logarithmic plot of the probability distribution function f(x) of the normalized distance *x* between two segments in a real topological polymer (the Kremer–Grest model) of N=300 with graph *F*. The fitted lines are given by $1.6\;x^{0.7}$, $1.1\;x^{0.7}$, $1.2\;x^{0.9}$ and $1.4\;x^{1.15}$ for a linear (red circles), a ring (green upper triangles), a theta curve (light blue stars), and a complete bipartite graph $K_{3,3}$ polymer (dark blue squares), respectively. Reprinted with permission from [31].

The probability distribution function f(x) of the normalized distance *x* between two segments in a real topological polymer (the Kremer–Grest model) of N=300 with graph *F* is plotted in the double logarithmic scale in Figure 10 for each of the four graphs such as linear, ring, θ-shaped and complete bipartite $K_{3,3}$ graphs.

The enhancement of the short-distance correlation is characterized by the different values of the exponent for the short-distance power-law behavior. It is given by 0.7 for both a linear and a ring polymer, while 0.9 and 1.15 for a theta curve polymer and a complete bipartite graph $K_{3,3}$ polymer, respectively, as shown in Figure 10.

For star polymers, the Kratky plot of the static structure factor is calculated for real chains [159]. It seems, however, that the large-*q* behavior is similar to that of an open chain.

## 5. The Probability of Random Knotting

### 5.1. Definition of the Knotting Probability

We now define the probability of random knotting for a model of random polygons (RP) or self-avoiding polygons (SAP) of *N* nodes. Suppose that we have an algorithm in the model by which we can generate an ensemble of RP or SAP of *N* nodes. We define the random knotting probability of a knot *K* for the model by the probability that a polygon of *N* vertices generated by the algorithm has the given knot type *K* [64,65,66]. We also call it the knotting probability of a knot *K*. We denote it by $P_{K}\left(N\right)$.

For lattice polygons, the total number of polygons of *N* nodes, Z(N), is finite. Then, we denote by $Z_{K}\left(N\right)$ the total number of polygons of *N* segments with a given knot type *K*. It is clear that $Z_{K}\left(N\right)<Z\left(N\right)$. The probability for a RP or SAP with *N* segments having the knot type *K* is given by:

(41) $$P_{K}\left(N\right)=Z_{K}\left(N\right)/Z\left(N\right)\;.$$

The knotting probability $P_{K}\left(N\right)$ of a knot *K* depends on the model of RPs or SAPs. However, the *N*-dependence of the knotting probability has some universal properties, which do not change for different models of random polygons or self-avoiding polygons.

### 5.2. Experiments Measuring the Knotting Probability

The knotting probabilities have been measured in experiments. The knotting probability of the trivial knot, that of the trefoil knot and even those of a few nontrivial knots were measured in the experiments of randomly closing nicked circular DNA chains, where the two ends of the corresponding linear DNA chains are connected by controlling the temperature of the solution [34,35]. The experimental results are compared with the theoretical values of the knotting probabilities for the model of worm-like circular chains.

Quite recently, the knotting probability of very long circular DNA, such as 166 kbp, has been measured successfully in a solid-state nanopore experiments [161]. We also remark that ring polymers of some nontrivial knot were synthesized by chemical reactions [57].

### 5.3. Numerical Method for Evaluating the Knotting Probabilities

Let us explain the method for how we evaluate the knotting probabilities of several knots, numerically. Suppose that a large number of RPs or SAPs is generated by an algorithm of a given random model. We denote by $W_{all}$ the number of all of the generated polygons. For each of the generated polygons, we calculate some knot invariants, such as the Vassiliev invariants, and then select such polygons that have a given knot type *K* [64,66,68]. If a polygon has the same set of values of the knot invariants as the knot *K*, then we assume that the topology of the polygon is given by the knot *K*. Here, the number of selected polygons, $W_{K}$, can be much smaller than that of all polygons, $W_{all}$. By the ratio $W_{K}/W_{all}$, we evaluate the knotting probability for knot *K*, $P_{K}\left(N\right)$.

We often employ the Vassiliev invariant of the second degree and the determinant of a knot as the tool for detecting the knot type. Here, we remark that for the definition of the knot invariants; see, for instance, the textbook [162]. With the values of the two knot invariants, we detect the knot type of the polygon (see Table 3) [66,68]. The Vassiliev invariants can be calculated in polynomial time by the method of the R-matrix [64,66] or by the method of the Gauss diagrams [163].

We remark that the present method for evaluating knotting probabilities gives practically accurate values for them. There might be a certain complex knot that has the same set of values of the knot invariants employed in the method. However, the number of wrong identifications of knot types should be small if the number of nodes *N* is not very large or knot types are not very complex.

### 5.4. Cylindrical SAPs as the Discrete Worm-Like Chains

In the review, we present our simulation results of the discrete worm-like chain model [34,35] for ring polymers in good solution. The model produces an ensemble of SAPs consisting of impenetrable cylinder-shape segments with unit length and cylindrical radius $r_{\mathrm{ex}}$ [81]. The cylindrical SAP model is suitable for studying statistical properties of circular DNA chains where the long-range Coulomb interactions are screened by counter ions in solution.

**Figure 11:**
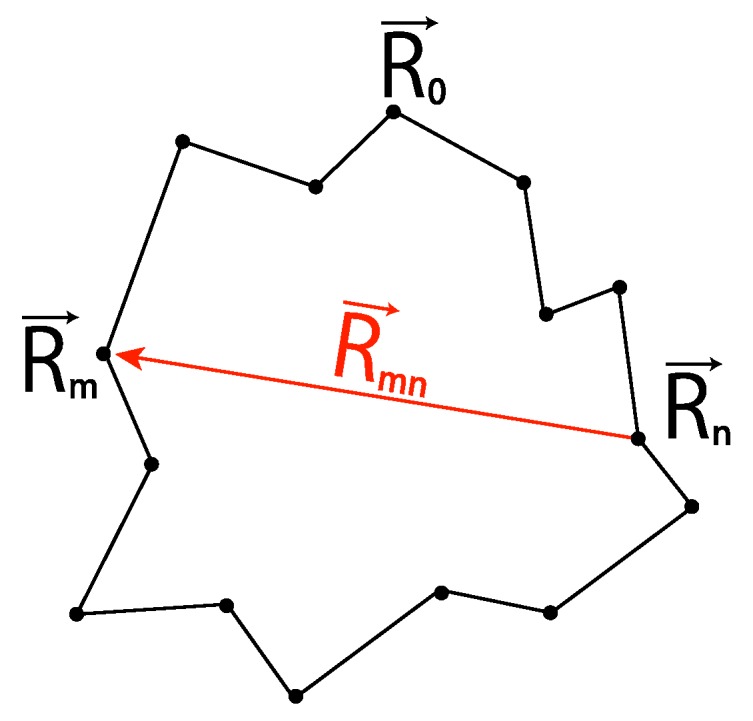
Random polygon (or self-avoiding polygon) of *N* nodes corresponds to a conformation of a ring polymer chain in solution consisting of *N* statistical segments.

The conformation of a cylindrical SAP is given by an equilateral polygon of *N* vertices (Figure 11). The excluded volume is assigned by the condition that the distance between any pair of non-neighboring segments in the polygon is larger than $2r_{\mathrm{ex}}$: each polygonal edge has unit length, and non-neighboring cylindrical segments do not overlap, while neighboring segments may overlap.

We generate an ensemble of SAP of *N* hard cylindrical segments by applying the MC procedures: We choose two nodes of a given polygon randomly and rotate one of the sub-chains between them around the line connecting them by a random angle and check whether there is any overlap among the cylindrical segments or not. We employ the conformation after applying 2N MC procedures and add it to an ensemble of the cylindrical SAP. The precise algorithm for generating cylindrical SAPs is formulated in [81].

In the case of $r_{\mathrm{ex}}=0$, the SAPs generated by the above algorithm are given by equilateral random polygons. Here, we recall that they have no excluded volume. The algorithm for generating cylindrical SAPs with $r_{\mathrm{ex}}=0$ is also called the polygonal folding method (PFM) [164]. The ergodicity of PFM is shown in [137,164] (see also [165]).

### 5.5. Formula for the *N*-Dependence of the Knotting Probability

It was shown numerically that the *N*-dependence of the knotting probability of a knot *K* is well approximated by the following formula [67]:

(42) $$P_{K}\left(N\right)=C_{K}{\left(\frac{N-\Delta N(K)}{N_{K}}\right)}^{m(K)}\exp\left(-\frac{N-\Delta N(K)}{N_{K}}\right)\;.$$

There are four fitting parameters: $N_{K},\;C_{K},\;m\left(K\right)$ and ΔN(K). We call m(K) and $C_{K}$ the exponent and the coefficient of the knotting probability of knot *K*, respectively. It was shown that the four-parameter formula (42) gives good fitted curves to the simulation data of the knotting probabilities of several knots as functions of *N* with respect to the $\chi^{2}$ values [67,68], where the random knotting probabilities of several knots were evaluated for the Gaussian random polygon and the rod-bead model with several different values of bead radius [67]. Recently, it was shown that Formula (42) gives good fitted curves for 145 different knots [81] (see also [166]).

**Figure 12:**
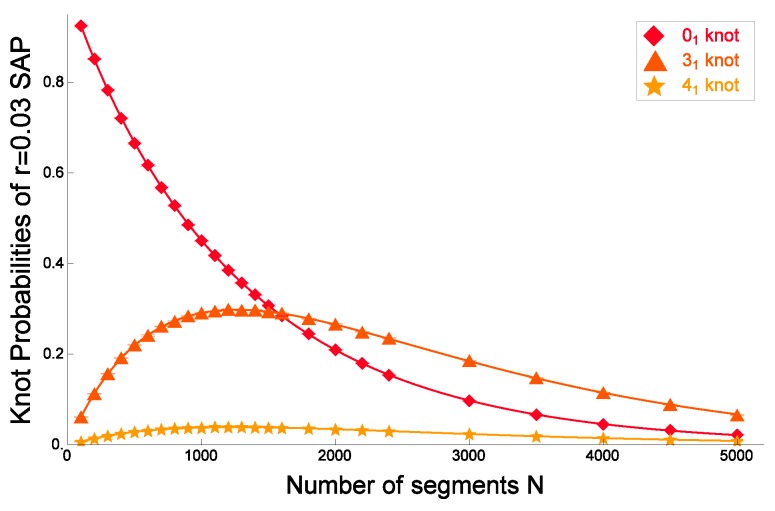
Knotting probabilities of the trivial ($0_{1}$), the trefoil ($3_{1}$) and the figure-eight ($4_{1}$) knots versus the number of segments *N* for cylindrical SAP with cylindrical radius $r_{\mathrm{ex}}=0.03$. The knotting probabilities of knots $3_{1}$ and $4_{1}$ for the Gaussian random polygons with *N* up to 1600 are depicted in [66] with less good statistics.

As shown in Figure 12 and Figure 14, Formula (42) gives good fitted curves to the knotting probabilities of some prime knots and composite knots for the cylindrical SAP with radius $r_{\mathrm{ex}}=0.03$.

The four-parameter Formula (42) generalizes the exponential decay of the knotting probability of the trivial knot to the case of nontrivial knots. In previous numerical studies [61,62,63], it was found that the knotting probability of the trivial knot $0_{1}$, which we also call the unknotting probability, decays exponentially with respect to the number of segments *N*, i.e., $m(0_{1})=0$ in Formula (42).

(43) $$P_{0_{1}}\left(N\right)\approx C_{0}\exp\left(-N/N_{0}\right)\;.$$

The exponential decay was shown for the rod-bead model with different values of bead radius [63]. It was shown in numerical simulation [65] that the knotting probability of a nontrivial knot has a maximal value as a function of *N*.

Formula (42) for the knotting probability of a knot *K* is similar to the asymptotic expansion of the logarithm of the partition function of the knotted ring polymer with knot *K* [66]. However, this is not necessarily the case. If we neglect the parameter ΔN(K) of finite-size correction, Formula (42) corresponds to the asymptotic expansion of the partition function $Z_{K}\left(N\right)$ with respect to the number of segments *N*.

(44) $$\log\left(Z_{K}\left(N\right)/Z\left(N\right)\right)=-\frac{N}{N_{0}}+m\left(K\right)\log N/N_{0}+\log C_{K}+O\left(1/N\right).$$

From the renormalization-group viewpoint [130], we suggest that the exponent m(K) should be given by a universal value for each knot *K* [67]. For SAP on the square lattice, it is suggested that the exponents for the asymptotic expansion m(K) should be given by integers at least approximately [69,70]. In fact, it is consistent with the additivity of exponents m(K)’s, as shown in Equation (46). However, for RPs or SAPs with finite numbers of segments *N* are concerned, the best estimates of m(K) are different from integers, particularly if we introduce the cut-off parameters ΔN(K) [67].

The fitting parameters ΔN(K) give cut-offs in the range of small *N* values for the knotting probabilities $P_{K}\left(N\right)$. The introduction of the cut-off parameters ΔN(K) improves the $\chi^{2}$ values by taking into account finite-size corrections.

The introduction of the parameter ΔN(K) is favorable to the mathematical result on the smallest number of segments for an SAP with a given knot to exist. It was shown rigorously that the knot type of an SAP in the cubic lattice is given by the trivial knot if the number of segments *N* is smaller than 24: N<24 [120]. Therefore, the knotting probability of the trefoil knot $\left(3_{1}\right)$ for SAP of *N* vertices vanishes if N<24 for SAP on the cubic lattice. The theorem was extended also for other knots [121].

We remark that the ratios between knotting probabilities of two different knots are studied for lattice polygons for asymptotically large *N* [78].

### 5.6. Unknotting Probability versus Excluded Volume

We now argue that the knotting probability of any given nontrivial knot for a fixed number of segments *N* decreases if the excluded volume increases.

We call the fitting parameter $N_{0}$ the characteristic length of random knotting. The estimates of the parameters $N_{K}$ are given by almost the same value with respect to statistical errors for several different knots [67]. Moreover, it is shown that it is the case for the 145 different knots [81]. Here, the suffix 0 of $N_{0}$ denotes the trivial knot, $0_{1}$.

The explicit value of $N_{0}$ depends on the model of random polygons or self-avoiding polygons. The estimate of $N_{0}$ is approximately given by 300 for random polygons such as Gaussian random polygons and equilateral random polygons [67]. For self-avoiding polygons on lattices, the characteristic length $N_{0}$ becomes quite large, such as the order of $10^{5}$ [62,73].

We recall the discrete worm-like chain model for ring polymers in good solution [34,35]. The model produces an ensemble of SAPs consisting of impenetrable cylinder-shape segments of unit length with cylindrical radius $r_{\mathrm{ex}}$ [81]. For the model, the characteristic length $N_{0}$ as a function of cylindrical radius $r_{\mathrm{ex}}$ is evaluated as [71]:

(45) $$N_{0}\left(r_{\mathrm{ex}}\right)=N_{0}\left(0\right)\exp\left(\alpha r_{\mathrm{ex}}\right)\;.$$

Here, the parameters are estimated as $N_{0}\left(0\right)=292\pm 5$ and α=43.5±0.6 [71]. We remark that a more precise approximation of the characteristic length $N_{0}$ as a function of cylindrical radius $r_{\mathrm{ex}}$ is given in [81].

It follows from exponential decay (43) and exponential growth (45) that the unknotting probability approaches 1.0 for any number of segments *N* if the excluded volume increases. Here, we recall the exponential decay of the unknotting probability (43) and the exponential growth of characteristic length $N_{0}$ with respect to cylindrical radius. If the cylindrical radius $r_{\mathrm{ex}}$ increases, then the characteristic length $N_{0}$ becomes very large, and hence, the knotting probability of the trivial knot, $P_{0_{1}}\left(N\right)$, for an SAP with *N* segments approaches 1.0. Therefore, the knotting probabilities of nontrivial knots become very small for a fixed number of segments *N* if the cylindrical radius becomes large.

### 5.7. Random Knotting Probabilities of Composite Knots

**Figure 13:**
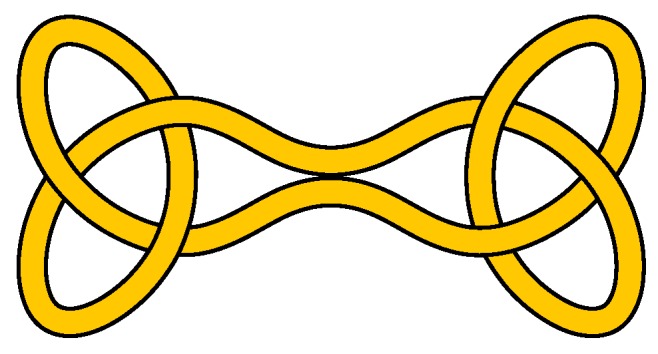
Knot diagram of a composite knot consisting of two trefoil knots ($3_{1}\#3_{1}$).

We denote by $K_{1}\#K_{2}$ the composite knot for a given pair of knots $K_{1}$ and $K_{2}$. For an illustration, a composite knot consisting of two trefoil knots is shown in Figure 13.

It has been observed numerically for the first time in the Gaussian random polygons that the exponents m(K) of knots *K* satisfy the additivity for composite knots [66]:

(46) $$m\left(K_{1}\#K_{2}\right)=m\left(K_{1}\right)+m\left(K_{2}\right)\;.$$

Coefficients $C_{K}$s have an empirical property. From the list of the best estimates of $C_{K}$’s [67], we observe that the estimates of $C_{K}$ for composite knots satisfy the factorization property [77,79]:

(47) $$C_{K_{1}\#K_{2}}=C_{K_{1}}C_{K_{2}}\;.$$

We denote the *n*-fold composite knot of a knot *K* by $K^{\#n}$. For instance, we have $K^{\#n}=K{\left(\#K\right)}^{n-1}=K\#K\#\cdots \#K$. For an *n*-fold composite knot $K^{\#n}$ we have the factor of the inverse of the *n*-factorial, i.e., n!:

(48) $$C_{K^{\#n}}={\left(C_{K}\right)}^{n}/n!\;.$$

The factorization property for the knotting probability of a composite knot has been found for SAP on the cubic lattice through simulation [76].

**Figure 14:**
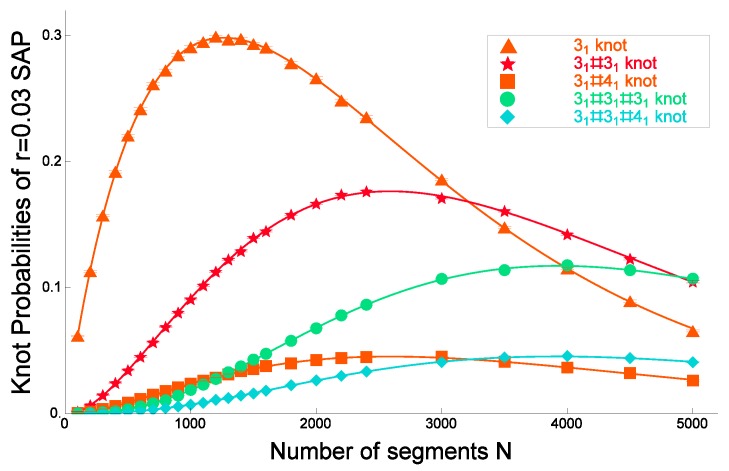
Knotting probabilities of the trefoil knot ($3_{1}$) and its composite knots $3_{1}\#3_{1}$, $3_{1}\#4_{1}$, $3_{1}\#3_{1}\#3_{1}$ and $3_{1}\#3_{1}\#4_{1}$ with versus the number of segments *N* with good fitted curves for cylindrical self-avoiding polygons (SAP) with cylindrical radius $r_{\mathrm{ex}}=0.03$. The knotting probabilities of composite knots such as $3_{1}\#3_{1}$, $3_{1}\#4_{1}$ and $3_{1}\#3_{1}\#3_{1}$ are plotted for the Gaussian random polygons with *N* up to 1600 in less good statistics in [66].

In Figure 14, the knotting probabilities of the trefoil knot $3_{1}$, its composite knots $3_{1}\#3_{1}$, $3_{1}\#4_{1}$, $3_{1}\#3_{1}\#3_{1}$ and $3_{1}\#3_{1}\#4_{1}$ are plotted against the number of segments *N* for cylindrical SAP with cylindrical radius $r_{\mathrm{ex}}=0.03$. Here, the fitted curves are given by applying Formula (42) to the data points. They are good since they have small $\chi^{2}$ values per degree of freedom. The best estimates of the fitted curves in Figure 12 and Figure 14 are given in [166]. For different models of RP and SAP, such as the Gaussian random polygons and the rod bead model, similar plots are given in [67]. Here, we also recall Figure 3.2 of [66].

We now argue that Formula (42) gives good fitted curves to the data points of the knotting probabilities of various knots versus the number of segments *N* with respect to the $\chi^{2}$ values. In fact, we have applied Formula (42) to the data of the knotting probabilities of 145 different knots versus the number of segments *N* for ten different values of the cylindrical radius in the cylindrical SAP model (i.e., the discrete worm-like chain model for ring polymers in good solution) [81].

We suggest that the additivity of exponents (46) and the factorization property of coefficients (47) should be valid also for other models of random polygons or self-avoiding polygons. Then, we can estimate the knotting probability of a composite knot $K_{1}\#K_{2}$ by making use of the additivity of exponents (46) and the factorization property of coefficients (47) from the knotting probabilities of constituent knots $K_{1}$ and $K_{2}$ for various models of random polygons or self-avoiding polygons.

## 6. Scaling Behavior of Random Knots

### 6.1. Mean-Square Radius of Gyration for RP and SAP under Topological Constraints

Let us consider a model of RP or SAP of *N* segments. Hereafter, we denote the mean-square radius of gyration of RP or SAP with a fixed knot type *K* by ${\langle R_{g}^{2}\rangle }_{K}$ or simply by $R_{g,K}^{2}$ instead of ${\langle R_{G}^{2}\rangle }_{K}$ expressed in Equation (1). We denote by $R_{g,all}^{2}$ the mean-square radius of gyration for random polygons of *N* nodes under no topological constraint [131]. Here, all denotes that all possible polygons of *N* nodes under no topological constraint.

Let us recall that symbol ${\langle A\rangle }_{K}$ denotes the ensemble average of a physical quantity *A* over all possible configurations of random polygons with a given knot *K*. We now explain the method for how we calculate ${\langle A\rangle }_{K}$, numerically. We first construct a large number of random polygons or self-avoiding polygons of *N* segments. To each of them, we calculate the two knot invariants, the Vassiliev invariant of the second degree and the determinant of knots. We select such polygons that have the same set of values of the two knot invariants as knot *K*. We calculate the average value of *A* over the selected polygons.

Let us review briefly the asymptotic behavior of the average size of polymer chains with excluded volume [167,168,169,170,171]. According to the RG arguments [168], it is predicted that the mean-square radius of gyration $\langle R_{g}^{2}\rangle$ of any polymer chain should have the asymptotic behavior:

(49) $$\langle R_{g}^{2}\rangle =A_{R_{g}}N^{2\;\nu_{\mathrm{SAW}}}\left(1+b_{R_{g}}^{\left(1\right)}N^{-\Delta_{1}}+\cdots \right),$$

as *N* increases infinitely [169]. The critical exponents $\nu_{\mathrm{SAW}}$ and $\Delta_{1}$ are universal. The estimates $\nu_{\mathrm{SAW}}=0.5880\pm 0.0015$ and $\Delta_{1}=0.470\pm 0.025$ were obtained by RG arguments through the n=0 limit of the *n*-vector field theory model [167], while the estimates $\nu_{\mathrm{SAW}}=0.5877\pm 0.0006$ and $\Delta_{1}=0.56\pm 0.03$ were obtained by the simulation of three-dimensional SAWs on lattice [169].

For asymptotically large *N*, we expect that the mean-square radius of gyration for SAP with fixed knot *K* is given by:

(50) $$R_{g,K}^{2}=A_{K}N^{2\nu_{K}}\left(1+O(1/N^{\Delta })\right),$$

where $A_{K},\nu_{K}$ and Δ are fitting parameters. We now define the effective scaling exponent. Suppose that we determine the parameter $\nu_{K}$ through the fitted curves derived by applying Equation (50) to the data of $R_{g,K}^{2}$ versus the segment number *N*. We then call it the effective scaling exponent. We expect that the effective scaling exponent $\nu_{K}$ for SAP with fixed knot *K* is given by that of SAW: $\nu_{\mathrm{SAW}}=0.588$ [130].

### 6.2. Topological Swelling of Random Knots

The mean-square radius of gyration of random polygons or self-avoiding polygons under a topological constraint of being equivalent to a knot *K*, denoted by $R_{g,K}^{2}$, can become significantly larger than that of no topological constraint $R_{g,all}^{2}$. We call the phenomenon topological swelling.

Topological swelling occurs even for self-avoiding polygons if the excluded-volume parameter is small. Let us consider cylindrical self-avoiding-polygons (SAP) consisting of cylindrical segments. Here, each of the segments is given by a cylinder of unit length whose radius is given by $r_{\mathrm{ex}}$. The mean-square radius of gyration was evaluated for cylindrical SAPs in simulation [85,86]. It was then shown that topological swelling occurs if the value of *r* is very small, such as 0.01. Recently, it has been reported that the topological swelling is observed in experiments of synthetic ring polymers [95].

According to des Cloizeaux [43], the topological constraints on ring polymers in the θ solution should play a similar role as the excluded volume. The suggestion has been studied by several authors through numerical simulations [82,88,89,90,91].

**Figure 15:**
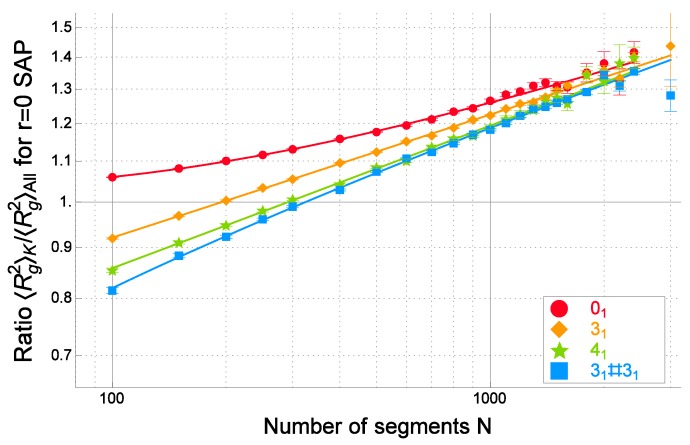
Topological swelling of equilateral random polygons with fixed knot: Double logarithmic plots of the ratio of the mean-square radius of gyration of equilateral random polygon with a fixed knot *K* to that of no topological constraint, ${\langle R_{g}^{2}\rangle }_{K}/{\langle R_{g}^{2}\rangle }_{\mathrm{All}}$ versus the number of segments *N* up to N=3000 for the trivial knot ($0_{1}$), the trefoil knot ($3_{1}$), the figure-eight knot $(4_{1}$) and the composite knot ($3_{1}\#3_{1}$). For the Gaussian random polygons with *N* less than 2000 the ratio is plotted against *N* in the double-logarithmic scale for knots $0_{1}$, $3_{1}$ and $4_{1}$ in [88] with less good statistics.

Let us consider the average size of ring polymers in a θ solvent. Here, the θ temperature is given by that of linear polymers corresponding to the ring polymers, where the excluded-volume interaction among segments is effectively screened. From the graphs of $3_{1}$ (${\langle R_{g}^{2}\rangle }_{3_{1}}/{\langle R_{g}^{2}\rangle }_{\mathrm{All}}$) and $4_{1}$ (${\langle R_{g}^{2}\rangle }_{4_{1}}/{\langle R_{g}^{2}\rangle }_{\mathrm{all}}$) of Figure 15, we observe that for a nontrivial knot, the average size, $R_{K}$, is smaller than that of no topological constraint, $R_{all}$, if *N* is small, such as $N<N_{0}$. However, it becomes larger than $R_{all}$ if *N* is large enough, such as $N\gg N_{0}$.

### 6.3. Effective Scaling Exponent of RP with Fixed Knot

The mean square radius of gyration $R_{g,K}^{2}$ of random polygons with fixed knot *K* has been numerically evaluated in some models for a few knots, and the effective scaling exponent $\nu_{K}$ has been numerically estimated [82,88,89,90,91]. In [82,89,90,91] the numerical estimates of the effective scaling exponent $\nu_{K}$ are definitely larger than $\nu_{\mathrm{RP}}=0.5$, i.e., the scaling exponent of random polygons under no topological constraint. In fact, in [89,90,91], the estimates are given by the scaling exponent of self-avoiding walks, $\nu_{\mathrm{SAP}}=0.588$, while in [88], the estimate is close to the scaling exponent of random walks, $\nu_{\mathrm{RP}}=0.5$.

It is not trivial to determine the value of the effective scaling exponent $\nu_{K}$ through numerical simulation. In the previous simulations, the number of polygonal nodes *N* is not very large: we have N≤2400 for all of the simulations performed so far [82,88,89,90,91]. Thus, the range of *N* is not large enough to determine the value of the scaling exponent $\nu_{K}$. If the range of *N* is not wide enough, it is possible that the same data points are compatible with two different values of the effective scaling exponent ν as far as the $\chi^{2}$ values are concerned [90]: one is close to 0.5 and another close to 0.6.

Quite recently, for the cylindrical SAP model, we have determined the effective scaling exponent by making use of good fitted curves to the data of the ratio ${\langle R_{g}^{2}\rangle }_{K}/{\langle R_{g}^{2}\rangle }_{\mathrm{All}}$ not only as a function of the number of segments *N*, but also as a function of the excluded volume parameter [166]. Through the plot of the effective scaling exponent $\nu_{K}$ for SAP with knot *K* versus the excluded volume parameter, we evaluate the effective scaling exponent of RP with fixed knot *K*. The value is larger than 0.5 and smaller than 0.588 with respect to errors. It increases very slowly as the upper limit of the range of the number of segments *N* increases. Detailed results will be given in a subsequent paper [166].

## 7. Conclusions

We have shown various aspects of topological polymers as polymers with different structures in chemical connectivity, as well as polymers with nontrivial topology, such as knotted ring polymers. If the structure in chemical connectivity or the topology of the polymer is nontrivial, there are many aspects to be studied through simulation and theoretical approaches.

For instance, we have reviewed in Section 3.1 that if all of the vertices are trivalent, the effect of excluded volume near the vertices is not relevant to the mean-square radius of gyration or the hydrodynamic radius for the topological polymer. We have argued in Section 3.3 that the ratio $R_{G}/R_{H}$ of a given polymer is approximately determined by the ratio of the variance to the mean square of the distance between two segments in the polymer. It supports the universality of the ratio $R_{G}/R_{H}$. Moreover, we have reviewed that the ratio $R_{G}/R_{H}$ of a topological polymer is expressed in terms of the pair distribution function (the two-point correlation function). We have then discussed the knotting probability of RP and SAP, i.e., fundamental aspects of ring polymers with nontrivial topology in Section 5. We have also argued possible asymptotic behavior of RP and SAP with fixed knot in Section 6.

The contents of the present review should be useful for making further progress in the numerical and theoretical studies of topological polymers.

## Figures and Tables

**Table 1:** Ratio of coefficients, $a_{1}\left(F\right)/a_{1}\left(\mathrm{ring}\right)$, in Equations (3) and (4) for ideal and real topological polymers with graphs *F*, respectively.

Graph *F*	Ratio of $a_{1}$ (Ideal)	Ratio of $a_{1}$ (Real)
linear	1.978±0.015	1.847±0.038
tadpole (lasso)	1.368±0.009	1.340±0.018
ring	1.000±0.003	1.000±0.007
double-ring	0.753±0.002	0.850±0.002
θ-shaped	0.667±0.004	0.699±0.003
complete $K_{3,3}$	0.413±0.002	0.4857±0.0005

**Table 2:** Ratio of the gyration radius to the hydrodynamic radius $R_{G}/R_{H}$ versus the number of segments *N* for ideal and real topological polymers with graph *F*.

Graph *F*	*N*	$R_{G}\left(F\right)/R_{H}\left(F\right)$ (Ideal)	$R_{G}\left(F\right)/R_{H}\left(F\right)$ (Real)
linear	500	1.425±0.025	1.47±0.42
tadpole (lasso)	500	1.380±0.021	1.354±0.072
ring	500	1.253±0.013	1.205±0.027
double-ring	500	1.215±0.011	1.190±0.032
θ-shaped	500	1.194±0.013	1.112±0.017
complete $K_{3,3}$	501	1.132±0.031	1.043±0.014

**Table 3:** Values of the two knot invariants for some knots: the determinant of a knot (the Alexander polynomial evaluated at t=−1, $\Delta_{K}\left(t=-1\right)$) and the Vassiliev invariant of the second degree, $v_{2}\left(K\right)$, for a knot type *K*.

Knot Type	$0_{1}$	$3_{1}$	$4_{1}$	$5_{1}$	$5_{2}$	$3_{1}\#3_{1}$	$3_{1}\#4_{1}$
Determinant of knot	1	3	5	5	7	9	15
2nd order Vassiliev invariant	0	1	−1	3	2	2	0
